# Genome-wide association study reveals candidate genes associated with egg-laying performance in Wuhua yellow chicken

**DOI:** 10.1016/j.psj.2025.105739

**Published:** 2025-08-26

**Authors:** Xunhe Huang, Zhipeng Zhong, Zhifeng Zhang, Zhuoxian Weng, Yongjie Xu, Weina Li, Guohao Zhong, Qing Wang, Yufei Shi, Tingting Xie, Li Zhang, Cheng Ma, Bingwang Du

**Affiliations:** a Guangdong Provincial Key Laboratory of Conservation and Precision Utilization of Characteristic Agricultural Resources in Mountainous Areas, Guangdong Innovation Centre for Science and Technology of Wuhua Yellow Chicken, School of Life Sciences, Jiaying University, Meizhou, 514015, China; b College of Coastal Agricultural Sciences, Guangdong Ocean University, Zhanjiang, Guangdong, 524088, China; c Department of Medical Biochemistry and Microbiology, Uppsala University, Uppsala, Sweden

**Keywords:** Genetic parameter, Egg production, Clutch size, Age at first egg, Wuhua yellow chicken

## Abstract

Egg production traits are economically critical in poultry farming. However, the genetic mechanisms underlying these traits in indigenous chicken breeds remain largely unknown. In this study, we conducted a genome-wide association study (**GWAS**) using whole-genome sequencing data from 315 Wuhua yellow chickens, an indigenous breed characterized by low egg production but considerable genetic diversity. Phenotypic assessments included age at first egg (**AFE**), egg number (**EN**), and clutch size traits across three laying stages. The SNP-based heritability estimates ranged from 0.10 to 0.38, with AFE showing negative genetic correlations with EN and clutch-related traits. We identified 871 significant SNPs (51 genome-wide and 820 suggestive) associated with egg production traits and annotated 379 candidate genes. This study revealed that *SCUBE1* and *KRAS* are important regulators of AFE through follicular development and metabolic pathways. Notably, *IGF1* and *PTK2* are associated with clutch size and EN, primarily through the mTOR and insulin signaling pathways. Additionally, 13 quantitative trait loci (**QTLs**) overlapped with known reproductive loci, including *SOX5* and *PPFIBP1*. Functional enrichment analyses underscored the significant involvement of the identified genes in cell adhesion, hormone signaling, and oocyte maturation pathways. These findings improve our understanding of the genetic architecture of egg production traits in indigenous chickens and highlight potential molecular targets for marker-assisted selection to enhance egg yield while preserving genetic diversity.

## Introduction

Egg production traits, such as age at first egg (**AFE**), egg number (**EN**), and clutch size, are economically important for poultry farming and directly influence profitability (Gautron et al., 2022; Yang et al., 2023). These traits exhibit polygenic inheritance with low-to-moderate heritability and are influenced by both genetic and environmental factors, making their genetic architecture complex and challenging to dissect (Zhang et al., 2012; Qin et al., 2015). Although commercial breeds, such as Rhode Island Red and White Leghorn, produce approximately 300 eggs annually, indigenous Chinese chickens typically yield less than 150 eggs (China National Commission of Animal Genetic Resources, 2011). This difference in productivity highlights the importance of understanding the genetic factors that influence egg production in diverse populations.

Advances in genomic technologies have enabled the identification of genetic markers associated with egg production traits. Genome-wide association studies (**GWAS**) have revealed numerous candidate genes, including *VTG2* and *PDCD11* for EN in Laiwu Black chickens (Lei et al., 2024); *SCUBE1, IGF2BP1*, and *GIP* for AFE (Chen et al., 2024; Ma et al., 2024); and *PTK2* for clutch size (Volkova et al., 2025). The Chicken QTLdb currently catalogs 29,116 quantitative trait loci (**QTLs**) related to economic traits, including 637 for EN and 119 for to AFE (https://www.animalgenome.org/cgi-bin/QTLdb/GG/index, accessed on June 14, 2025). Notably, whole-genome sequencing approaches have identified key genomic regions, such as a 0.57 Mb segment on GGA10 containing *NEO1* and *ADPGK* in Shuanglian chickens (Fu et al., 2023). These findings demonstrate the complex genetic architecture underlying egg production traits.

The Wuhua yellow chicken, an indigenous breed from Guangdong Province, China, represents a valuable yet understudied genetic resource. Previous studies have established foundational knowledge regarding genetic diversity, evolutionary history, and candidate genes associated with plumage coloration (Huang et al., 2020; Weng et al., 2020, 2022). Despite its distinctive yellow plumage and superior meat quality, the breed displays low annual egg production (typically < 120 eggs; ∼85 eggs as documented in the Poultry Genetic Resources in China; Chen et al., 2004) but considerable phenotypic variation in reproductive traits. Unlike intensively selected commercial lines, the Wuhua yellow chicken retains a largely unselected genetic background, making it particularly valuable for identifying novel genetic variants associated with egg production traits.

In this study, we aimed to characterize the genetic architecture of egg production traits in Wuhua yellow chickens through GWAS analysis; identify candidate genes and pathways associated with AFE, EN, and clutch size traits; and compare these findings with known QTLs in chicken breeds. These results provide crucial insights for enhancing egg production in indigenous chicken populations and maintaining their valuable genetic diversity.

## Materials and methods

### Ethics statement

All animal experiments were approved by the Institutional Animal Care and Use Committee of Jiaying University (protocol number: JYDWLL2024-12). All procedures were performed in accordance with the committee guidelines.

### Animals and phenotypic data collection

In this study, 315 Wuhua yellow hens from Fengdu Breeding Farm (Meizhou, China) were used. The chickens were raised in group housing and transferred to individual cages at 14 weeks of age. All chickens were managed according to the standard protocols for Wuhua yellow chickens. The egg production traits were recorded daily from the first egg until 46 weeks of age. We classified four egg production traits, namely, AFE, EN, average clutch size (**ACS**), and maximum clutch size (**MCS**). A clutch was defined as consecutive days of egg laying without a break. ACS was calculated as the mean number of eggs per clutch, whereas MCS represented the maximum number of consecutive eggs laid during specific periods. Based on the characteristic trends observed in the laying curve of our Wuhua yellow chicken population, we divided the laying period into three phases (Fig. S1) and counted the EN, ACS, and MCS in each phase: the early laying stage with a rapid increase in egg production (laying rate < 40 % before 21 weeks of age) (EN20 w); peak laying stage (laying rate > 60 % from 21 to 30 weeks of age) (EN21–30 w , ACS21–30 w, and MCS21–30 w); declining laying stage from 31 to 46 weeks of age (EN31–46 w, ACS31–46 w, and MCS31–46 w) was subdivided based on the characteristic average laying rate into a phase typically around 55–60 % (EN31–38 w, ACS31–38 w, and MCS31–38 w) and a subsequent phase where the average rate fell below 55 % (EN39–46 w, ACS39–46 w, and MCS39–46 w). We also calculated the total EN (**ENT**), total ACS (**ACST**), and total MCS (**MCST**) from the first eggs to 46 weeks of age.

### DNA extraction and genome resequencing

Blood was collected from the wing veins of 315 hens at 47 week of age. Genomic DNA was extracted using a HiPure Universal DNA Kit (Magen Biotechnology, China) following the manufacturer’s protocol. DNA quality was assessed using 1 % agarose gel electrophoresis and quantified using a NanoDrop 2000 spectrophotometer (Thermo Fisher Scientific, USA). DNA libraries were constructed with an insert size of approximately 350 bp using an MGIEasy PCR-Free DNA Kit (MGI Tech Co., Ltd., Shenzhen, China). Libraries were sequenced on the DNBSEQ-T7 platform (Annoroad, China) to generate 150 bp paired-end readso at ∼10 × coverage.

### Genotyping and quality control

Raw reads were processed using Fastp v0.20.0 to remove adapters and filter low-quality reads (Chen, 2023). Clean reads were aligned to the chicken reference genome (GRCg6a, GCA_000002315.5) using BWA-MEM v0.7.17 with the default parameters (Li and Durbin, 2010). After alignment, the reads were sorted, and duplicates were marked and removed using Picard Tools v3.3.0. Variant calling was performed using GATK HaplotypeCaller v4.2.0.0 (Mckenna et al., 2010). Finally, SNPs were filtered using GATK VariantFiltration with options “–filter-expression” “QD < 2.0 || MQ < 40.0 || FS > 60.0 || QUAL < 30.0 || SOR > 3.0 || MQRankSum < −12.5 || ReadPosRankSum < −8.0″, and the output was further filtered using BCFtools v1.10.2 (Danecek et al., 2021). Data filtering steps were applied, including removing SNPs and individuals with missingness > 0.1, minor allele frequency < 0.01, and Hardy–Weinberg equilibrium *P*-value < 1.00E−6. Only the autosomal biallelic variants were retained for further analyses.

### Genetic parameter estimation

SNP-based heritability and pairwise genetic correlations of egg production traits were estimated using the restricted maximum likelihood (**REML**) approach implemented in GCTA v1.92.2 (Yang et al., 2011). The proportion of phenotypic variance explained by candidate SNPs and haplotypes was calculated using this method.

### Population genetics analysis

The whole-genome SNPs of all 315 individuals were pruned using PLINK v1.90 (Purcell et al., 2007) with the parameters (--indep-pairwise 50 10 0.1). Principal component analysis (**PCA**) of the pruned SNPs was conducted using the genetic relationship matrix generated by PLINK v1.90. These pruned SNPs were subsequently used for linkage disequilibrium (**LD**) decay analysis, with measurements calculated for pairwise SNPs using PopLDdecay v3.42 (Zhang et al., 2019).

### GWAS

SNP haplotypes were phased using BEAGLE v5.2 (Browning et al., 2018). All high-quality variants were subjected to additional pruning using the same parameters to obtain independent SNPs. Association analyses of egg production traits were conducted using a mixed linear model (**MLM**) in GEMMA v0.98.5 (Zhou and Stephens, 2012). The population stratification effect was corrected by incorporating the first three principal components (**PC**s) as covariates. To control for multiple testing, genome-wide significance thresholds were established at –log10(0.05/number of independent variants) for significant associations and –log10(1/number of independent variants) for suggestive associations. The GWAS results, including Manhattan and quantile-quantile plots, were visualized using the “CMplot” package (https://github.com/YinLiLin/CMplot) in R 4.4.2.

### Overlap with known QTLs

Genomic regions extending 100 kb upstream and downstream of each candidate SNP were examined for overlap with previously reported QTLs related to AFE, EN, egg production, and reproductive traits in chickens using Animal QTLdb (https://www.animalgenome.org/cgi-bin/QTLdb/GG/index; accessed 9 March 2025).

### LDBlock analysis and gene annotation

To characterize potential causal regions, LDBlock analysis was performed using LDBlockShow v1.40 (Dong et al., 2021) with all phased SNPs. Genes within the candidate regions were annotated using ANNOVAR v20240219 (Wang et al., 2010) and were considered potential candidate selected genes. Functional enrichment analyses, including Gene Ontology (**GO**) and Kyoto Encyclopedia of Genes and Genomes (**KEGG**) pathway analyses, were conducted using the KOBAS v3.0 online platform (Bu et al., 2021). Pathways with a *P*-value < 0.05 were considered significantly enriched.

### Statistical analysis

The association between SNP genotypes and phenotypic traits was assessed using a hypothesis-testing framework wherein, for each significant SNP, the appropriate statistical test was selected based on the distributional properties of the data. Firstly, the homogeneity of variances across genotype groups was verified using Levene's test. Subsequently, if the assumption of equal variances was met (*P* Levene > 0.05), differences between groups were assessed using one-way analysis of variance (ANOVA); if the assumption was violated (*P* Levene ≤ 0.05), the non-parametric Kruskal–Wallis test was employed. For analyses yielding a significant global effect (*P* < 0.05), pairwise comparisons were conducted using Tukey's Honestly Significant Difference (HSD) test following a significant ANOVA, or Dunn's test with Bonferroni correction following a Kruskal–Wallis test of significance. The specific test used for each genotypic association presented in the figures is indicated in the corresponding legends.

## Results

### Phenotypic and genetic parameter statistics

Summary statistics for egg production traits across laying stages are showed in Table 1. The AFE ranged from 114 to 185 d, with a mean of 130.18 d (approximately 19 weeks). The mean EN during the early laying phase (EN20w) was 24, whereas the mean ENT reached 110.77. The maximum values for both ACS and MCS occurred during the peak laying period (21–30 w), reaching 13.25 and 32, respectively.

**Table 1:** Descriptive statistics for egg production traits in different stages.

Traits	N	Min	Max	Mean	SD	CV (%)
AFE	315	114	185	130.18	12.01	9.23
EN20w	315	0	24	6.87	6.32	92.02
EN21–30w	315	8	63	43.33	10.33	23.85
EN31–38w	315	8	51	31.45	8.83	28.08
EN39–46w	315	0	47	29.12	8.97	30.78
EN31–46w	315	11	94	60.57	15.53	25.65
ENT	315	31	165	110.77	23.64	21.34
ACS21–30w	315	0	13.25	3.61	1.64	45.27
ACS31–38w	315	0	10.25	3.22	1.31	40.66
ACS39–46w	315	0	9.5	3.04	1.28	42.11
ACS31–46w	315	2	8.17	3.16	1.09	34.41
ACST	315	2	9.46	3.34	1.06	31.81
MCS21–30w	315	0	32	7.37	4.78	64.90
MCS31–38w	315	0	21	5.44	3.04	55.91
MCS39–46w	315	0	16	4.93	2.68	54.33
MCS31–46w	315	2	21	6.16	3.18	51.64
MCST	315	2	32	8.61	4.94	57.33

AFE: Age at first egg (days); EN: Egg number (w: weeks of age, with subscripts indicating age ranges); ENT: Total egg number; ACS: Average clutch size (subscripts indicate age ranges in weeks). ACST: Average clutch size from first egg to 46 weeks of age. MCS: Maximum clutch size (subscripts indicate age ranges in weeks). MCST: Maximum clutch size from first egg to 46 weeks of age.

**Fig. 1:**
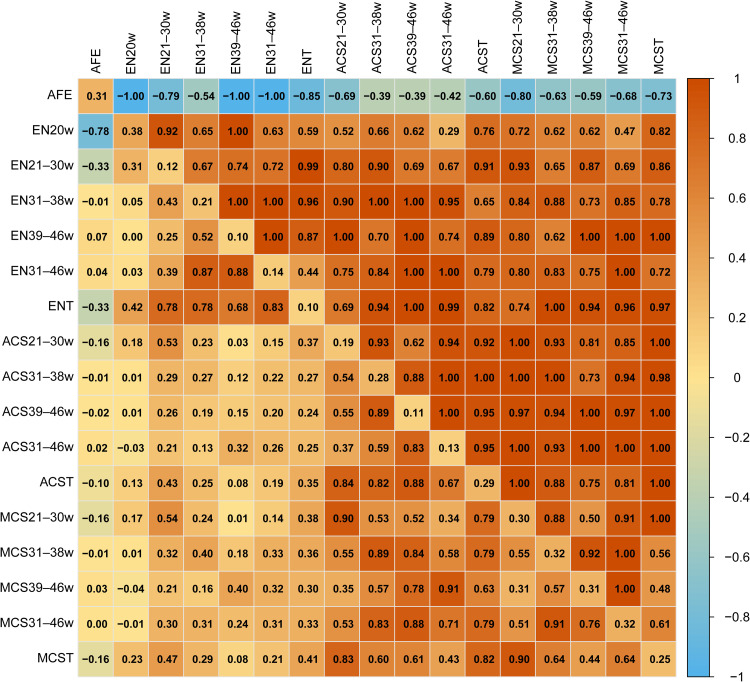
Heatmap of SNP-based heritabilities (on the diagonal), genetic (above the diagonal) and phenotypic (below the diagonal) correlations between egg production traits.

The SNP-based heritability estimates and genetic and phenotypic correlations for AFE, ENs, ACS, and MCS are summarized in Fig. 1. Heritability ranged from 0.10 to 0.38 across all 17 traits. A strong positive genetic correlation was observed between EN and clutch size. Notably, AFE showed negative genetic correlation coefficients with all other 16 traits. This pattern was also phenotypically evident, with AFE showing negative correlations with 9 of the 16 traits.

### Genome resequencing and variant analysis

Whole-genome resequencing of 315 Wuhua yellow chickens generated 4.44 Tb of high-quality data with an average sequencing depth of 12.18 × and coverage of 96.73 % (Table S1). After GATK quality control, we identified 17,483,435 clean SNPs, with most (>50 %) located in intronic regions, whereas only 1.82 % were exonic (Tables S2 and S3). Among the exonic variants, 2748 stop-gain, 271 stop-loss, and 125,644 nonsynonymous variants were identified (Table S3). This initial set was further refined by applying population-level filters, resulting in a final set of 11,263,512 high-quality SNPs which were used for GWAS. Following LD-based pruning, 306,083 independent SNPs were retained for subsequent analysis.

### Population genetics analysis

The PCA results revealed no significant population differentiation, confirming that the population structure would not confound association analyses (Fig. S2A). LD decay analysis showed rapid LD breakdown, potentially indicating limited selection pressure (Fig. S2B). The mean nucleotide diversity of the study population was 0.00397 ± 0.00020.

### Summary of GWAS

**Fig. 2:**
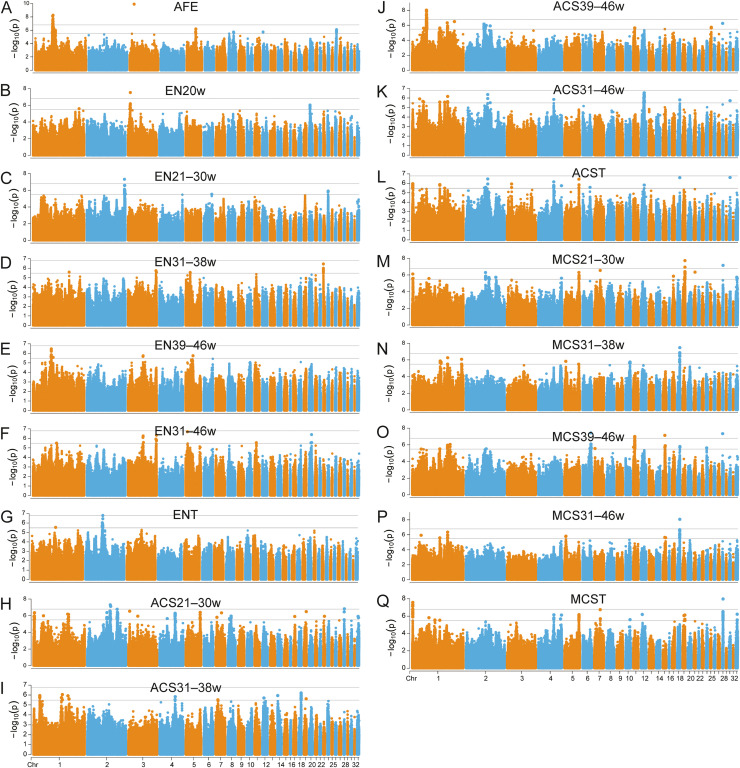
Manhattan plot of SNP-based genome-wide association signals for egg production traits in Wuhua yellow chicken. AFE represented age of first egg. EN20w, EN21–30 w, EN31–38 w, EN39–46 w, EN31–46 w represent egg number at 16–20 weeks of age, 21–30 weeks of age, 31–38 weeks of age, 39–46 weeks of age, 31–46 weeks of age, respectively. ENT represents total egg number. ACS21–30 w, ACS31–38 w, ACS39–46 w, and ACS31–46 w represent average clutch size in each of four stages consistent with the statistical stage division of egg number. ACST represents total average clutch size. MCS21–30 w, MCS31–38 w, MCS39–46 w, and MCS31–46 w represent maximum clutch size in each of four stages consistent with the statistical stage division of egg number. MCS represents maximum clutch size.

Our GWAS analyzed SNPs associated with AFE, EN, and clutch size (ACS and MCS) in 315 Wuhua yellow hens. Manhattan and Q-Q plots demonstrated well-controlled population structure, as evidenced by genomic inflation factors (λ) approximating 1 (Figs. 2, S3). We identified 871 genome-wide SNPs comprising 51 genome-wide significant (*P* < 1.63E−7) and 820 suggestive (*P* < 3.27E−6) variants distributed across 25 chromosomes, annotated to 335 and 44 genes, respectively (Table S4).

Among the annotated genes, 19 have been previously reported as candidate genes for egg production traits, including 11 for AFE, one for EN, three for ACS, and four for MCS (Table 2). These genes included basic helix-loop-helix family member e41 (***BHLHE41***) (Wang et al., 2024), capping actin protein of muscle Z-line alpha subunit 3 (***CAPZA3***) (Yuan et al., 2015), chitinase, acidic (***CHIA***) (Zhao et al., 2021), EPH receptor A1 (***EPHA1***) (Wang et al., 2024), homeodomain interacting protein kinase 2 (***HIPK2***) (Yuan et al., 2015), insulin-like growth factor 1 (***IGF1***) (Kim et al., 2004), PPFIA binding protein 1 (***PPFIBP1***) (Chen et al., 2024), SRY-box 5 (***SOX5***) (Ma et al., 2021), KRAS proto-oncogene, GTPase (***KRAS***) (Kabir et al., 2024), and vitamin K epoxide reductase complex subunit 1-like 1 (***VKORC1L1***) (Fu et al., 2023).

**Table 2:** Previous studies identified genes for egg production traits in chickens.

Traits^a^	Region	Gene name	Traits^b^	PMID
AFE	1:65799044	*SOX5*	Egg-laying rate	34102485
AFE	1:67193992–67204409	*KRAS*	Long-duration fertility	39311027
AFE	1:67537727–67585352	*RASSF8*	ACST	39152103
AFE	1:67572052–67585352	*BHLHE41*	ACS26–30w	39152103
AFE	1:68205138–68288365	*PPFIBP1*	AFE	38593551
AFE	1:68528880	*TSPO*	ACS26–30w	39152103
AFE	1:68575529–68692627	*SCUBE1*	AFE	38593551
AFE	1:68889440–68889701	*MPPED1*	AFE	38593551
AFE	1:78156269–78157329	*EPHA1*	ACS31–43w	39152103
AFE	8:20366141	*PTPRF*	EN35–48w	38598913
AFE	26:5081810	*CHIA*	Egg number	34376144
EN20w	3:7712575	*NRXN1*	EN4, TEN	35941697
ACS39–46w	1:55314100–55366596	*IGF1*	Egg production	15285513
ACS39–46w	1:56079196	*HIPK2*	EN37–72w	26496084
ACST	2:76099265	*ANKH*	Egg number	34376144
MCS21–30w, MCST	1:64306002	*CAPZA3*	Spermatid maturation	19341723
MCS21–30w	19:8974014–9119299	*MSI2*	AFE	38492250
MCS39–46w	24:2132541	*NTM*	AFE	31412760
MCST	19:5061159	*VKORC1L1*	EN43w	38136951

a Traits identified in this study.

b Traits reported in previous studies. EN4 (egg number from 308 to 354 d), TEN (total egg number from onset to 354 d).

### Comparing with previously reported QTLs

Using the Animal QTLdb, we detected 13 QTLs that overlapped with the significant SNPs identified in this study. Four of these QTLs were associated with AFE. The remaining nine QTLs were associated with egg production, including eight with EN and one with small yellow follicle number (Table S5). These 13 QTLs were linked to 13 candidate genes, seven of which have been previously reported to influence reproductive traits. These validated genes were *SOX5, PPFIBP1*, CUB domain, EGF-like domain containing 1 (***SCUBE1***), protein tyrosine phosphatase, receptor type F (***PTPRF***), *CHIA*, Musashi RNA binding protein 2 (***MSI2***), and *VKORC1L1*, all of which influence relevant biological pathways. Among these QTLs, significant SNPs for six QTLs, corresponding to *PPFIBP1, SCUBE1, PTPRF*, chondroitin sulfate synthase 1 (***CHSY1***), *VKORC1L1*, and *ENSGALG00000046550*, were located within the gene bodies, whereas the other seven contained significant SNPs in the upstream or downstream regulatory regions.

### Screening of SNPs associated with AFE

**Fig. 3:**
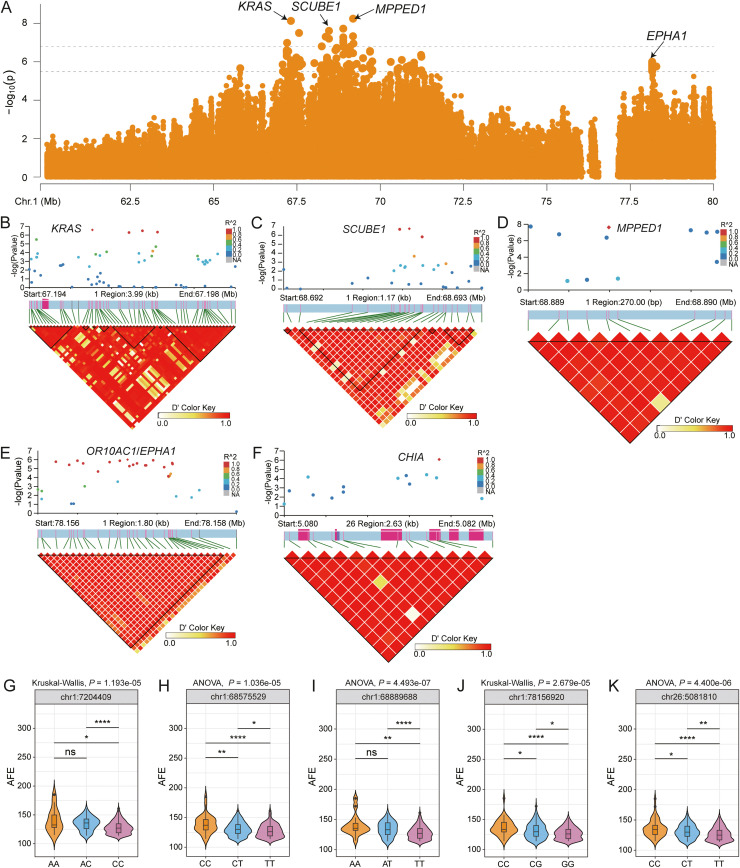
Identification of genetic loci that associate to age at first egg (AFE). (A) GWAS peak on chromosome 1. (B–E) Linkage disequilibrium analysis surrounding the peak on candidate genes (*KRAS, SCUBE1, MPPED1, EPHA1, CHIA*). (G–K) Genotype effects on AFE. (G,J) Data were analyzed by the Kruskal–Wallis test followed by Dunn's post-hoc test. (H,I,K) Data were analyzed by one-way ANOVA followed by Tukey's HSD post-hoc test. Significance levels are indicated as *P < 0.05, **P < 0.01, ***P < 0.001.

In total, 165 SNPs associated with AFE were distributed on chromosomes 1, 3, 5, 8, 12, and 26, corresponding to 63 annotated genes (Table S4). Chromosome 1 contained most SNPs (152/165) and genes (54/63). LDBlock analysis identified 120 SNPs forming LD blocks that were associated with 17 genes, including *SCUBE1*, Ras association domain family member 8 (***RASSF8***), *BHLHE41, SOX5*, glycogen synthase 2 (***GYS2***), *KRAS*, cytochrome b5 reductase 1 (***CYB5R3***), ADP ribosylation factor GTPase activating protein 3 (***ARFGAP3***), metallophosphoesterase domain containing 1 (***MPPED1***), and *EPHA1* on chromosome 1, along with *PTPRF* on chromosome 8, and *CHIA* on chromosome 26 (Fig. 3, Table S6).

Four distinct LD blocks were identified within or upstream of the *KRAS* gene region, all of which showed significant genotype-phenotype associations (Fig. 3A,B,G). Within the *SCUBE1* gene region, six distinct LD blocks were identified, including a prominent block comprising three tag SNPs representing 17 correlated SNPs (Fig. 3C). Notably, all eight *SCUBE1* SNPs showed significant genotype-phenotype associations (Fig. 3H). Similarly, five significant SNPs located within 10 kb downstream of the *MPPED1* gene formed an 11-SNP LD block, all of which demonstrated statistically significant associations (Fig. 3D,I). Additionally, a 28-SNP LD block was identified in the intergenic region between the olfactory receptor 10AC1 (***OR10AC1****)* and *EPHA1* (chr1:78156269–78157329), showing consistent significant associations in both phenotypic and genotypic analyses (Fig. 3E,J). Similarly, a 14-SNP LD block was observed in the *CHIA* gene, with the variant chr26:5081810 showing a significant genotype-phenotype association (Fig. 3F,K).

### Screening of SNPs associated with EN

**Fig. 4:**
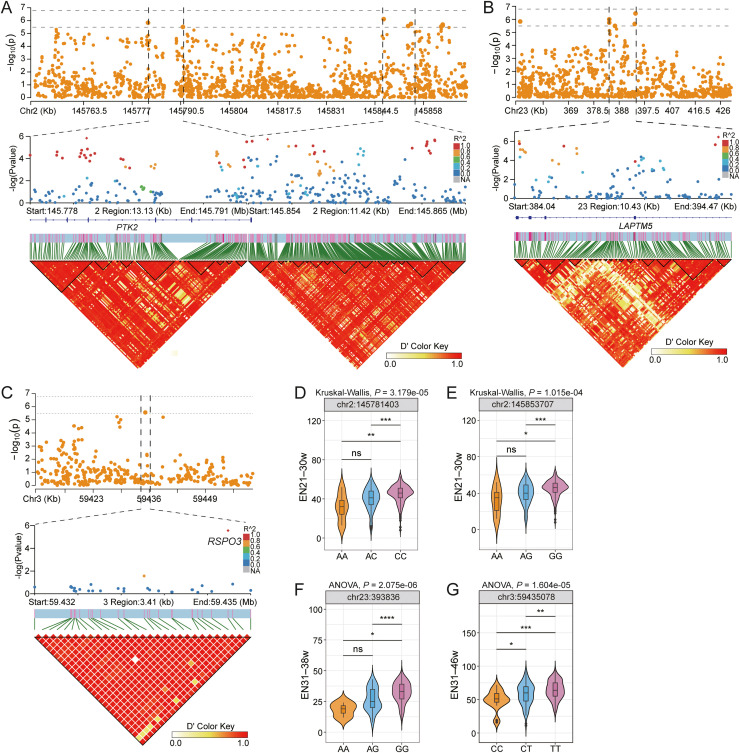
Identification of genetic loci that associate to egg number (EN). (A–C) Local Manhattan plot (top) and linkage disequilibrium analysis (bottom) surrounding the peak on candidate genes (*PTK2, LAPTM5, RSPO3*). (D–G) Genotype effects on EN. (D,E) Data were analyzed by the Kruskal–Wallis test followed by Dunn's post-hoc test. (F,G) Data were analyzed by one-way ANOVA followed by Tukey's HSD post-hoc test. Significance levels are indicated as *P < 0.05, **P < 0.01, ***P < 0.001.

For the ENs traits, 120 SNP candidate loci were identified and annotated to 70 genes (Table S4). Among these, 83 SNPs formed LD blocks associated with 45 genes, including protein tyrosine kinase 2 (***PTK2***), lysosomal protein transmembrane 5 (***LAPTM5***), and R-spondin 3 (***RSPO3***) (Fig. 4, Table S6). Within the *PTK2* gene region, seven SNPs formed five distinct LD blocks, including a prominent block comprising one tag SNP representing 40 correlated SNPs (Fig. 4A). Notably, all seven *PTK2* SNPs showed significant genotype-phenotype associations (Fig. 4D,E). In the *LAPTM5* gene, three distinct LD blocks were identified, with five variants demonstrating significant genotype-phenotype association (Fig. 4B,F). Additionally, we observed a significant SNP (chr3:59435078) located 35.6 kb downstream of *RSPO3*, which clustered within a 30-SNP block and showed a significant genotype-phenotype association (Fig. 4C,G).

### Screening of SNPs associated with ACS

**Fig. 5:**
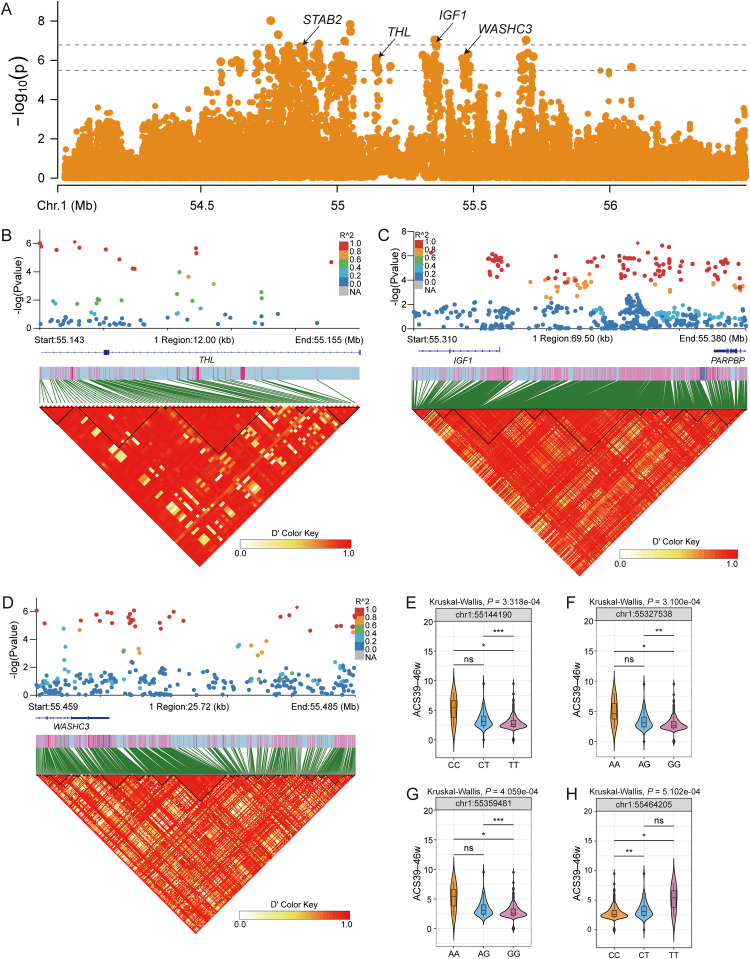
Identification of genetic loci that associate to average clutch size (ACS). (A) GWAS peak on chromosome 1. (B–D) Linkage disequilibrium analysis surrounding the peak on candidate genes (*THL, IGF, WASHC3)*. (E–H) Genotype effects on ACS. Data were analyzed by the Kruskal–Wallis test followed by Dunn's post-hoc test. Significance levels are indicated as *P < 0.05, **P < 0.01, ***P < 0.001.

For ACS traits, we identified 339 candidate SNP loci distributed across chromosomes 1–7, 11, 12, 14, 18, and 19, corresponding to 117 annotated genes (Table S4). The LDBlock analysis revealed that 265 SNPs formed LD blocks associated with 92 genes (Table S6). In the ACS39–46 w trait, we detected multiple genes within a 0.085 Mb region (5.548–5.563 Mb) on chromosome 1, including 5′-nucleotidase domain containing 3 (***NT5DC3***), stabilin 2 (***STAB2***), tyrosine hydroxylase (***THL***), *IGF1*, and WASH complex subunit 3 (***WASHC3***, also known as *CCDC53*). Additional genes associated with other ACS traits were identified on multiple chromosomes, including *HIPK2*, ANKH inorganic pyrophosphate transport regulator (***ANKH***)*,* and histone deacetylase 11 (***HDAC11***) (Fig. 5A).

The *THL* gene region contained seven SNPs forming four LD blocks, all of which showed significant genotype-phenotype associations (Fig. 5B,E). Genetic analysis of the *IGF1* gene identified 17 distinct LD blocks, which included a major block with 11 tag SNPs representing 148 correlated SNPs and nine additional blocks spanning over 10 kb each (Fig. 5C). Notably, all 11 tag SNPs demonstrated significant genotype-phenotype associations (Fig. 5F,G). Twelve SNPs in the *WASHC3* gene formed eight LD blocks, all of which exhibited significant genotype-phenotype associations (Fig. 5D,H). Additionally, the *STAB2* region contained 25 SNPs forming 17 LD blocks, all of which showed significant genotype-phenotype associations (Fig. S4).

### Screening of SNPs associated with MCS

For the MCS traits, we identified 247 candidate SNP loci annotated to 122 genes (Table S4). LDBlock analysis showed that 155 SNPs formed LD blocks associated with 79 genes, including solute carrier family 5 member 1 (***SLC5A1***), *HDAC11*, autophagy-related 14 (***ATG14***), regulatory associated protein of MTOR complex 1 (***RPTOR***), NCK adaptor protein 2 (***NCK2***), neurotrimin (***NTM***), and UBX domain protein 6 (***UBXN6***) (Table S6).

**Fig. 6:**
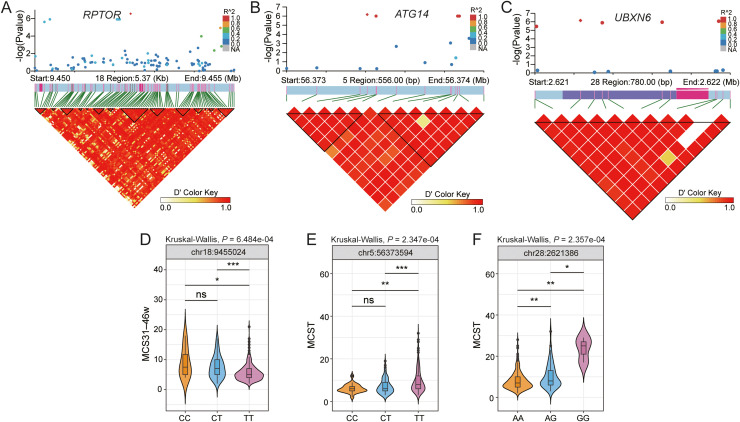
Identification of genetic loci that associate to maximum clutch size (MCS). (A–C) Linkage disequilibrium analysis surrounding the peak on candidate genes (*PRTOR, ATG14, UBXN6*). (D–F) Genotype effects on MCS. Data were analyzed by the Kruskal–Wallis test followed by Dunn's post-hoc test. Significance levels are indicated as *P < 0.05, **P < 0.01, ***P < 0.001.

Genetic association analysis revealed distinct patterns for *RPTOR, ATG14*, and *UBXN6* across the different phenotypic stages (Fig. 6). *RPTOR* was associated with both MCS31–38 W and MCS31–46 W, forming three LD blocks, with all SNPs showing significant genotype-phenotype association (Fig. 6A,D). *ATG14* was associated with MCS21–30 W and MCST, forming three LD blocks, with six SNPs showing significant genotype-phenotype association (Fig. 6B,E). Notably, *UBXN6* was specifically linked to MCST, with four SNPs forming a 12-SNP LD block, all four showed significant genotype-phenotype associations, including three located in the 3′ UTR region (Fig. 6C,F).

### Functional enrichment analyses of identified associated genes

**Fig. 7:**
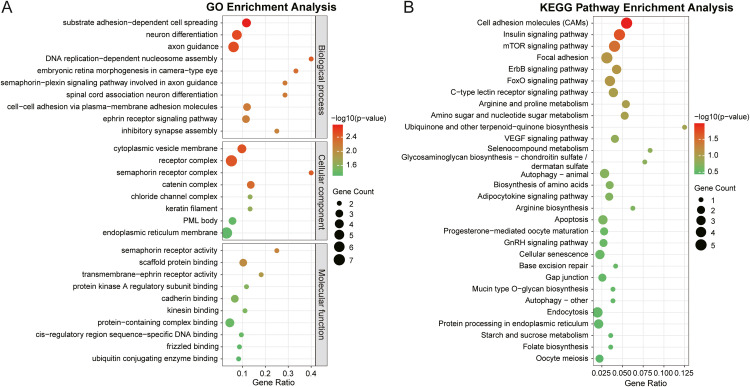
Functional enrichment analysis of 254 candidate genes. (A) GO enrichment analysis of candidate genes. Dot size corresponds to the number of enriched terms, and color represents the –log10(*P*-value) of the enriched terms, with a red-to-green gradient indicating –log10(*P*-value) ranging from large to small. (B) Top 30 of KEGG pathway analysis of candidate genes.

By integrating the gene lists from LDBlock analysis and published genes associated with egg production traits, we identified 254 candidate genes (Table S7). We performed GO enrichment and KEGG pathway analyses on the candidate genes. Fifty-one GO terms were enriched (*P* < 0.05) (Fig. 7A; Table S8), including 32 biological processes, seven cellular components, and 12 molecular functions. In the biological process category, four genes were enriched in substrate adhesion-dependent cell spreading, four in neuron differentiation, five in axon guidance, and four in DNA repair. For cellular components, 44 genes were enriched, including 31 in the integral component of the membrane, six in the receptor complex, and four in focal adhesion. Among the molecular functions, four genes were significantly enriched in protein-containing complex binding and 12 in identical protein binding.

GWAS identified 46 genes that were functionally enriched in 49 KEGG pathways, with five pathways showing enrichment (*P* < 0.05): mTOR signaling pathway, cell adhesion molecules pathway, insulin signaling pathway, focal adhesion, and ErbB signaling pathway (Fig. 7B; Table S9). Nine genes including *IGF1, CHIA, VKORC1L1, HIPK2, CAPZA3, PTPRF, MSI2*, translocator protein (***TSPO***), and neurexin 1 (***NRXN1***) were enriched in 11 pathways and have been verified to regulate egg production in chickens, including cell adhesion molecules (Wang et al., 2022), insulin signaling pathway (Lei et al., 2024), mTOR signaling pathway (Hao et al., 2021), amino sugar and nucleotide sugar metabolism (Zhao et al., 2021), ubiquinone and other terpenoid-quinone biosynthesis (Fu et al., 2023), progesterone-mediated oocyte maturation (Bhavana et al., 2022), cellular senescence (Sun et al., 2024), endocytosis (Yuan et al., 2015), adherens junction (Lei et al., 2024), mRNA surveillance pathway (Ma et al., 2024), AGE-RAGE signaling pathway in diabetic complications (Yan et al., 2022; Kabir et al., 2024), neuroactive ligand-receptor interaction (Ouyang et al., 2020), and metabolic pathways (Huang et al., 2022).

## Discussion

### Phenotypic and genotypic characteristics

Egg-laying performance is a key reproductive trait in chickens, and understanding its genetic basis is essential for improving low-productivity indigenous breeds. In the present study, we conducted a comprehensive analysis of multiple egg production traits in Wuhua yellow chickens. Our results revealed substantial variations in egg production. For example, AFE ranged from 114 to 185 d, ENT varied between 31 and 165 eggs, MCST showed a wide range from 2 to 32 d, and the coefficients of variation for these traits ranged from 9.23 % to 92.02 %. This pronounced phenotypic diversity likely indicates a lack of intensive selection in this indigenous population.

We calculated 17 genetic parameters for 315 hens. AFE showed moderate heritability (0.31), which is consistent with a previous finding (Ma et al., 2024). Existing studies exhibit variable heritability (0.19–0.62) depending on breed, population size, assessment methodology, and environment (Liu et al., 2019; Fu et al., 2023; Yang et al., 2023; Chen et al., 2024; Ma et al., 2024). The heritability of EN (0.10–0.38) was comparable to that observed by Chen et al. (2024) but lower than those reported by Wolc et al. (2019) and Ma et al. (2024). ACS (0.11–0.28) and MCS (0.25–0.32) heritability estimates were slightly lower than those reported in a previous study (Wang et al., 2024).

AFE showed consistent negative genetic correlations with EN, ACS, and MCS, whereas positive correlations were observed with the latter traits. Phenotypic correlations followed the same trend, which is consistent with previous findings (Fu et al., 2023; Ma et al., 2024).

### Genes associated with AFE

AFE is a key determinant of egg production potential. Our GWAS identified 165 AFE-associated SNPs corresponding to 63 genes, including *SCUBE1* and *MPPED1*, both of which have been previously implicated in AFE regulation (Chen et al., 2024) and were confirmed through QTL mapping. The *SCUBE1* gene encodes a secreted glycoprotein that mediates cell signaling and angiogenesis (Yang et al., 2002). Our analysis identified six LD blocks within *SCUBE1*, including two strong genotype-phenotype-associated SNPs (chr1:68575529 and chr1:68683679). Although known for its roles in mammalian bone formation (Lin et al., 2023), GO analysis implicated *SCUBE1* in chicken hedgehog signaling and the plasma membrane, suggesting potential roles in follicular development. These results established *SCUBE1* as a major AFE regulator, underscoring the need for further investigation of its ovarian expression profile.

Although both Chen et al. (2024) and our study identified significant associations between *MPPED1* variants and AFE, the underlying mechanism of this relationship remains elusive. The hydrolase activity of *MPPED1* suggests its potential involvement in AFE regulation via metabolic pathway modulation.

We identified three previously reported EN-related genes (*SOX5, PTPRF*, and *CHIA*) that also influence AFE. *SOX5* shows differential hypothalamic expression between high-yielding Rhode Island Red and low-yielding Lushi chickens (Ma et al., 2021), suggesting neuroendocrine regulation. Notably, Lei et al. (2024) recently identified *PTPRF* as being significantly associated with EN (EN35–48 w) through GWAS in Laiwu Black chickens, further supporting its involvement in follicular development through cell adhesion and insulin signaling pathways. Additionally, *CHIA* has been identified as a candidate gene in a GWAS of egg production traits (Zhao et al., 2021), potentially through its role in ovarian function and follicular development.

*EPHA1* emerged as a candidate gene associated with AFE. *EPHA1* receptor may modulate AFE through ephrin receptor signaling and integrin-mediated adhesion. *EPHA1* activation suppresses cell spread and migration via the ILK-RhoA-ROCK pathway (Yamazaki et al., 2009), potentially delaying follicular maturation or ovulation. Its transmembrane ephrin receptor activity and role in substrate adhesion-dependent cell spreading further substantiate its influence on ovarian function. A prominent LD block near *EPHA1* (chr1:78156269–78157329) with strong phenotypic links reinforced its genetic contribution to AFE.

Our study identified *KRAS* as significantly associated with AFE. This GTPase regulates reproductive function by modulating key signaling pathways. Through mTOR pathway activation, *KRAS* enhances glycolysis (Huang et al., 2021) and promotes lactic acid accumulation in the sperm storage tubules (Huang et al., 2021; Liu et al., 2023), a critical process for sperm quiescence maintenance (Matsuzaki et al., 2015). Concurrently, its participation in MAPK signaling preserves the oviduct epithelial barrier and optimizes sperm storage conditions. Proteomic evidence has demonstrated significant *KRAS* upregulation (*P* < 0.001) in high-fertility hens (Kabir et al., 2024), supporting its utility as a reproductive efficiency biomarker.

### Genes associated with EN

Several candidate genes identified by this GWAS have been previously associated with egg production, including *RSPO3* and *PTK2*, and our integrated analysis provides compelling evidence for *RSPO3*’s role in EN through multiple lines of investigation. The GWAS detected *RSPO3* within a prominent LD block containing 30 correlated SNPs, with the lead SNP chr3:59435078 showing strong genotype-phenotype associations. Functional annotation indicated that *RSPO3* is involved in key reproductive pathways, including glycosaminoglycan binding and Wnt signaling, consistent with its established function in sperm storage maintenance (Yang et al., 2020).

PTK2, also known as focal adhesion kinase (**FAK**), is a cytoplasmic non-receptor protein tyrosine kinase that regulates important reproductive and production traits across species, including egg-laying intervals in chickens (Volkova et al., 2025) and milk production in dairy cattle (Wang et al., 2013). Our GWAS and LDBlock analyses identified significant *PTK2* SNPs associated with egg production. Functional analyses indicated that *PTK2* participates in focal adhesion, ErbB signaling, and VEGF pathways. Mouse studies have demonstrated that *PTK2* is activated at oocyte–granulosa cell contact sites, where it stabilizes adherens junctions and promotes gap junction formation (McGinnis and Kinsey, 2015). Oocyte-specific *PTK2* knockout disrupts connexin-37/43 localization, reduces oocytes–granulosa cell communication, and impairs fertility, confirming its essential role in follicular function.

*LAPTM5* has been implicated in various cellular processes, including lysosomal function, membrane integrity, and protein degradation, as demonstrated by its classification in the lysosomal pathway and its role as an integral component of the membrane. The identified LD blocks and significant genotype-phenotype associations suggested *LAPTM5*’s potential involvement in EN traits, although additional functional validation is required.

### Genes associated with clutch size

Several genes identified in this GWAS are known candidates for egg production traits, including *IGF1, HIPK2, ANKH, CAPZA3*, pleckstrin homology domain containing A5 (***PLEKHA5***), *NTM, MSI2* and *VKORC1L1. IGF1* encodes a multifunctional polypeptide primarily secreted by the liver that regulates avian growth, skeletal development, and lipid metabolism (Kareem et al., 2016; Wang et al., 2021; Guo et al., 2023). *IGF1* is a key mediator of ovarian function, critically controlling follicular growth, differentiation, and maturation (Tosca et al., 2008; Wu et al., 2016). Importantly, *IGF1* influences key reproductive parameters, including ovulation rate, follicular development dynamics, and overall egg production capacity (Kim et al., 2004; Abdalhag et al., 2016; Liu et al., 2022; Uyanga et al., 2022; Xin et al., 2022). Genetic analyses identified 17 LD blocks in *IGF1*, including a key block with 11 tag SNPs representing 148 correlated SNPs. Functional analyses linked *IGF1* to crucial pathways (mTOR and insulin signaling pathways) and GO terms (phosphatidylinositol 3-kinase regulation and starvation response), confirming its nutrient-dependent reproductive role. Collectively, these findings establish *IGF1* as a master regulator of clutch size and reproductive efficiency in chickens.

Although direct evidence for *STAB2, THL*, and *WASHC3* in clutch size regulation is limited, our LDBlock analysis revealed distinct haplotype block structures with statistically significant genotype-phenotype associations. *STAB2* mediates placental reconstruction in mice (Kim et al., 2020), whereas *WASHC3* and *IGF1* co-participate in progesterone-mediated oocyte maturation. The genetic linkage among *STAB2, THL, IGF1*, and *WASHC3*, combined with *IGF1*’s established role in clutch size, strongly suggests that these genes may cooperatively regulate this reproductive trait through shared or interacting biological pathways.

Fu et al. (2023) identified *VKORC1L1* as being significantly associated with egg production at 43 weeks, which was supported by QTL co-localization. Although no LD block was formed in this study, chr19:5061159 showed a significant genotype-phenotype association. Functional analyses revealed *VKORC1L1*’s involvement in oxidative stress response, membrane integrity, ubiquinone biosynthesis, and metabolic pathways, as well as potential mechanisms affecting egg production through antioxidant protection and energy metabolism.

The RNA-binding protein *MSI2* plays a critical role in reproductive development (Arumugam et al., 2010). Although associated with AFE in chickens (Ma et al., 2024), our study revealed a significant association with MCS21–30 w (notably, genotype chr19:8975111 AA vs. AG). Functional enrichment linked *MSI2* with mRNA surveillance and protein binding, suggesting translational control mechanisms. These findings align with those of mammalian studies demonstrating *MSI2*’s essential role in follicle development and showing impaired folliculogenesis, reduced oocyte quality, and compromised fertility (Sutherland et al., 2015), underscoring its conserved involvement in reproductive efficiency.

Other identified reproduction-associated genes include *HIPK2* (Yuan et al., 2015), *ANKH* (Zhao et al., 2021), *CAPZA3* (Yuan et al., 2015), and *NTM* (Liu et al., 2019). *CAPZA3* regulates spermatid maturation through F-actin organization with mutations causing male sterility (Geyer et al., 2009). *RPTOR* (mTORC1 component) and *ATG14* (autophagy regulator) influence egg production via metabolic and developmental pathways. *RPTOR* modulates follicular development via mTOR and insulin signaling (Gorre et al., 2014; Yang et al., 2025), whereas *ATG14* affects primordial germ cell formation (Ding et al., 2024). Both intersect with TGF-β/BMP signaling, highlighting their importance for egg quality and ovarian function (Ding et al., 2024; Yang et al., 2025). *UBXN6* may regulate clutch size through post-transcriptional mechanisms, as suggested by its association with a 12-SNP LD block containing three significant 3′UTR variants. Functional studies indicated *UBXN6*’s role in endoplasmic reticulum-associated degradation and autophagy (Kim et al., 2024), which are critical for cellular homeostasis and reproductive physiology.

## Conclusions

This study presents the first comprehensive genome-wide association analysis of egg production traits in Wuhua yellow chickens. Our results demonstrated substantial phenotypic variation and moderate heritability, indicating the breed’s unselected genetic background. The identification of novel candidate genes (*SCUBE1, KRAS*, and *EPHA1*) that regulate AFE through the hypothalamic-pituitary-gonadal axis function enhances our understanding of sexual maturation in poultry. For clutch traits, our findings confirm the conserved role of *IGF1* in follicular development as well as reveal its specific genetic variants associated with consecutive laying performance in this indigenous breed. The identification of 13 significant SNPs overlapping with known QTLs but showing unique allele frequencies in Wuhua yellow chickens highlights both shared and breed-specific genetic architectures underlying reproduction. Pathway analyses revealed the importance of mTOR signaling and focal adhesions in reproductive efficiency. These findings provide valuable genetic insights and a molecular basis for improving egg production in indigenous chickens using targeted breeding strategies. Future studies should focus on functional validation, including transcriptomic analyses of reproductive tissues to elucidate the roles of candidate genes and their associated pathways in regulating egg-laying performance.

## Funding

This work was supported by the Key Discipline Construction Project of Guangdong Provincial Department of Education (2022ZDJS088), the Peak Talent Program of Jiaying University (2022RC45), the Scientific Research Innovation Team Project of Jiaying University (2021JYUTDS04), and the Open Fund Project of Guangdong Provincial Key Laboratory of Conservation and Precision Utilization of Characteristic Agricultural Resources in Mountainous Areas (2023JYKF06).

All new sequencing data generated in this study have been deposited in the NCBI sequence read archive (SRA) under BioProject ID PRJNA1288062.

## CRediT authorship contribution statement

**Xunhe Huang:** Writing – review & editing, Writing – original draft, Software, Project administration, Methodology, Investigation, Funding acquisition, Formal analysis, Data curation, Conceptualization. **Zhipeng Zhong:** Writing – review & editing, Software, Resources, Formal analysis, Data curation. **Zhifeng Zhang:** Software, Resources, Data curation. **Zhuoxian Weng:** Resources, Data curation. **Yongjie Xu:** Resources, Data curation. **Weina Li:** Resources, Data curation. **Guohao Zhong:** Software, Resources, Data curation. **Qing Wang:** Resources, Data curation. **Yufei Shi:** Resources, Data curation. **Tingting Xie:** Writing – review & editing, Funding acquisition. **Li Zhang:** Writing – review & editing. **Cheng Ma:** Writing – review & editing. **Bingwang Du:** Writing – review & editing, Resources.

## Disclosures

The authors declare that they have no known competing financial interests or personal relationships that could have appeared to influence the work reported in this paper.

## Appendix Supplementary materials

Image, application 1

Image, application 2

Image, application 3

Image, application 4

Image, application 5

Image, application 6

Image, application 7

Image, application 8

Image, application 9

Image, application 10
