# Supplementary material for: Genome-wide association study reveals candidate genes associated with egg-laying performance in Wuhua yellow chicken
Source: Poult Sci. 2025 Aug 26;104(11):105739. doi: 10.1016/j.psj.2025.105739 (PMC12414907; doi:10.1016/j.psj.2025.105739)
Supplement: Supplementary file 1 [file mmc1.docx]

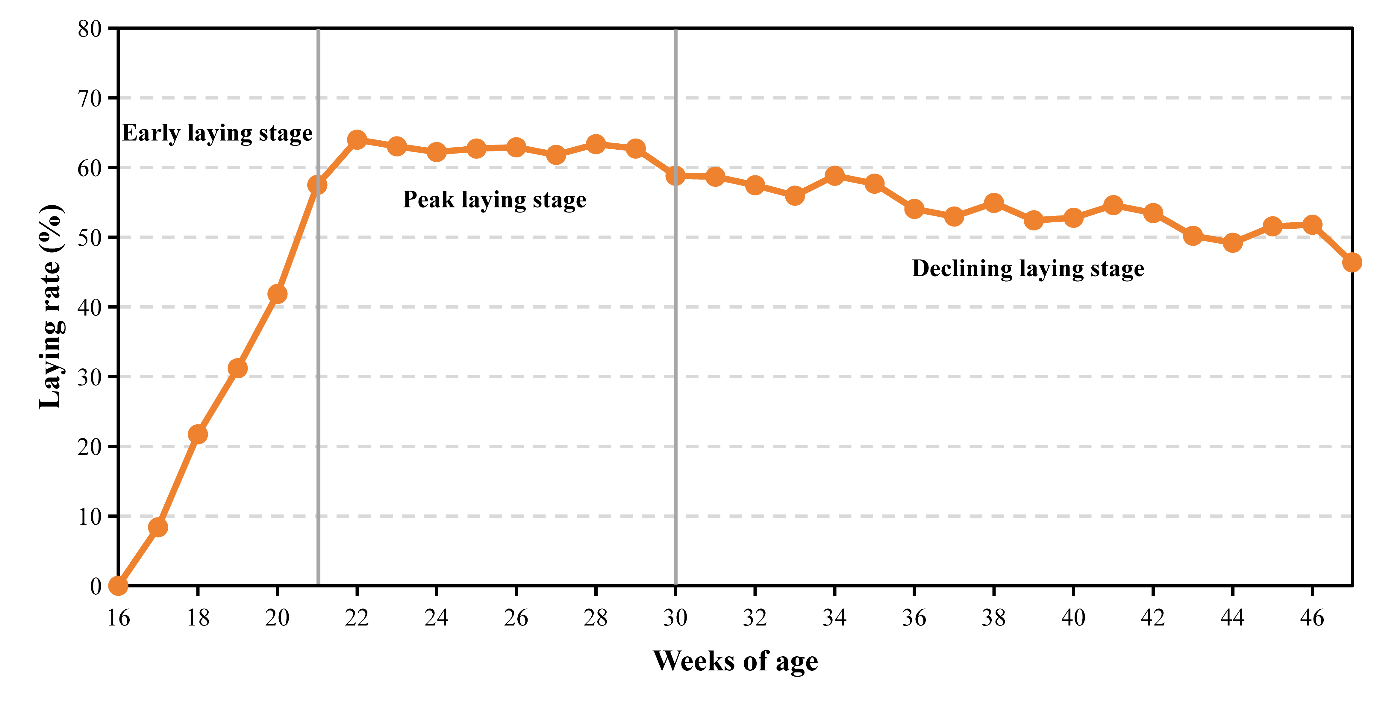


**Figure S1.** Heatmap of SNP-based heritabilities (on the diagonal) and genetic (above the diagonal) and phenotypic (below the diagonal) correlations between egg production traits.


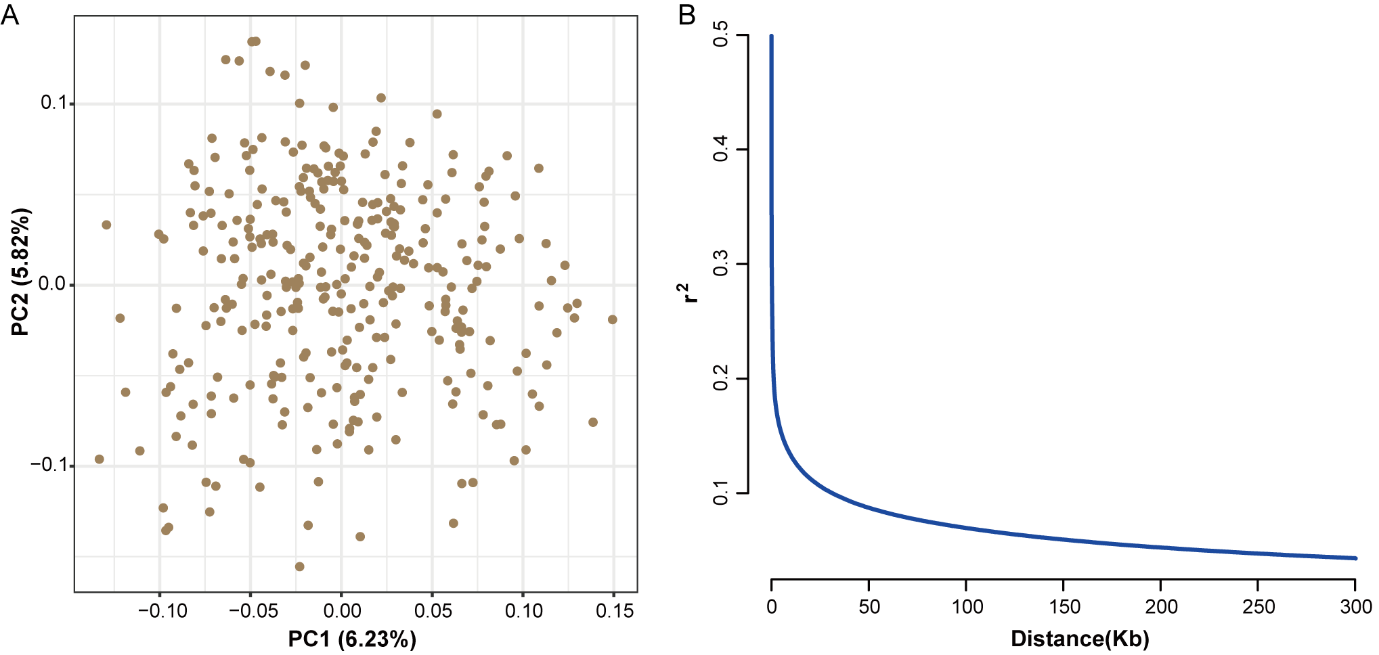


**Figure S2.** The Principal component plot and LD decay for 315 individuals.


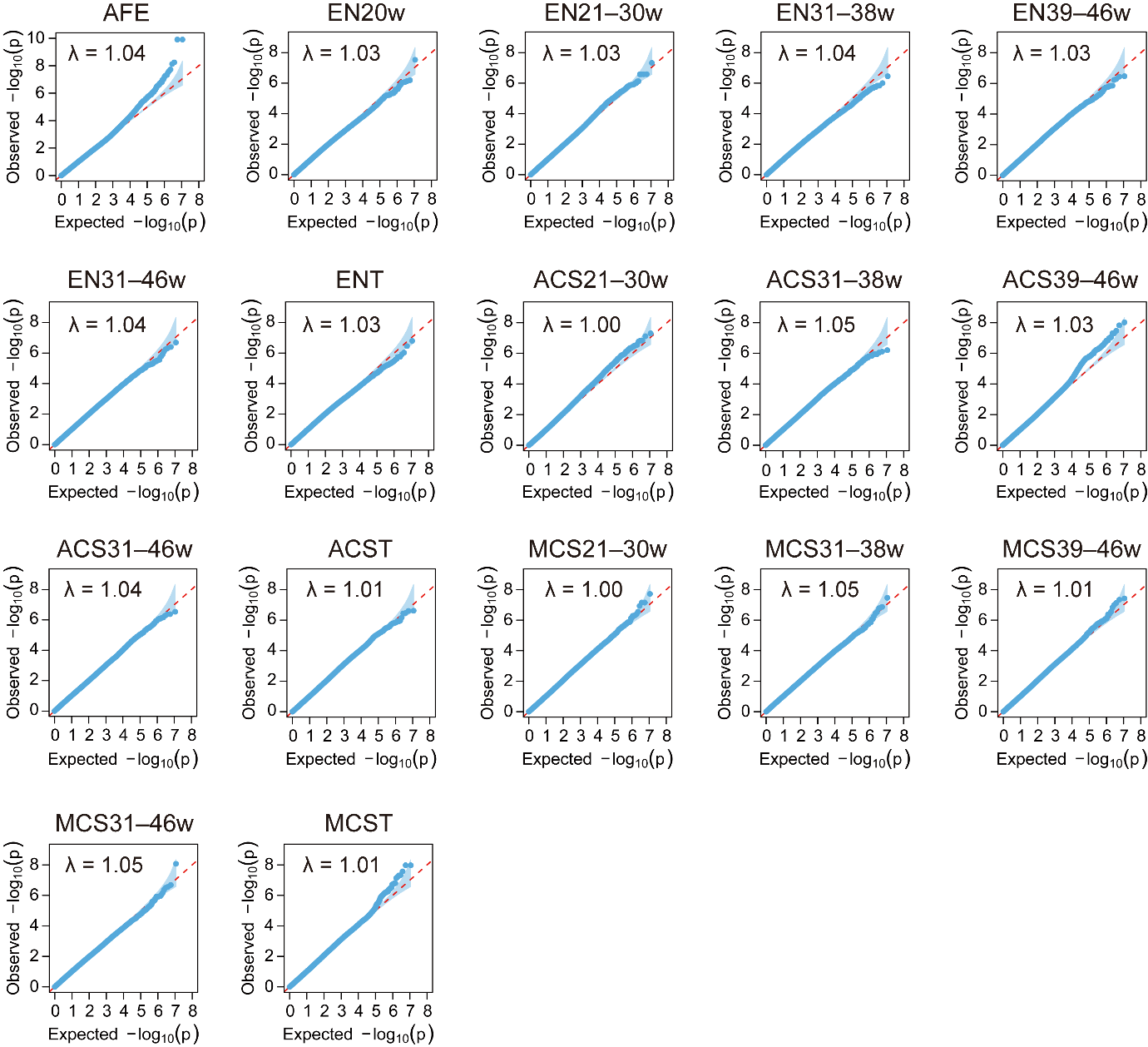


**Figure S3.** Quantile-quantile (Q-Q) plots of GWAS signals for egg production traits in Wuhua yellow chicken. Q-Q plots were displayed as scatter plots of observed and expected log *P*-values.


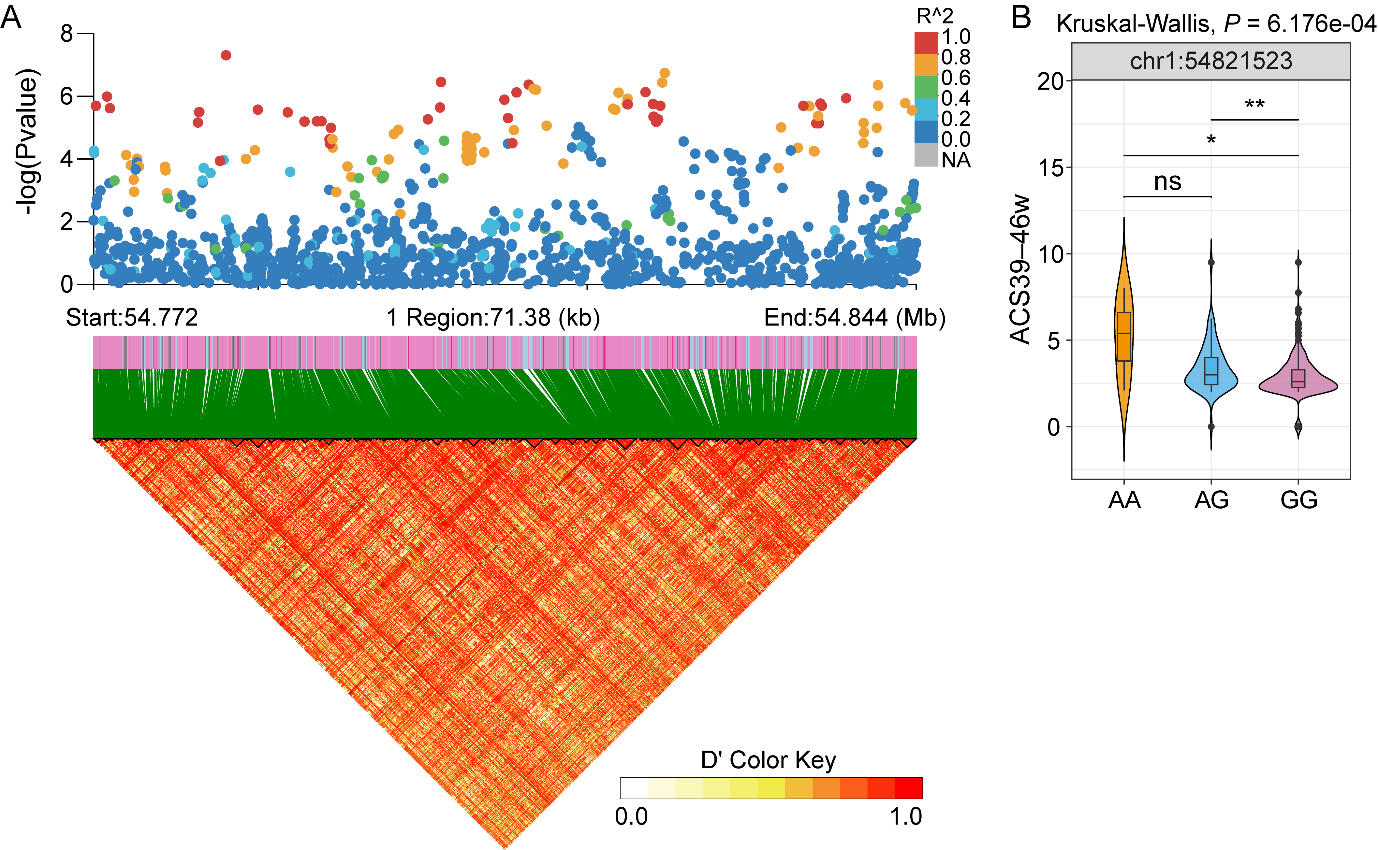


**Figure S4.** Identification of genetic loci that associate to MCS. (A) Linkage disequilibrium analysis surrounding the peak on candidate gene *STAB2*. (B) Genotype effects on MCS. Data were analyzed by the Kruskal–Wallis test followed by Dunn's post-hoc test. Significance levels are indicated as **P* < 0.05, ***P* < 0.01, ****P* < 0.001.
